# Supplementary material for: Interventions for Promoting Meconium Passage in Very Preterm Infants—A Survey of Current Practice at Tertiary Neonatal Centers in Germany
Source: Children (Basel). 2022 Jul 27;9(8):1122. doi: 10.3390/children9081122 (PMC9406488; doi:10.3390/children9081122)
Supplement: Supplementary file 1 [file children-09-01122-s001.zip › children-1821591-supplementary.pdf]

DEBUG ▶ Fragebogen [UMF_MEK] – Seite 1 ◀ Seite: ▶ Debug-Informationen ▼
Variable: = ▶

Universitätsklinikum Tübingen

0% ausgefüllt

A101

Maßnahmen zur Mobilisierung von Mekonium bei Frühgeborenen

Erhebung der aktuellen Praxis an Level 1-Perinatalzentren in Deutschland

Es ist derzeit unklar, ob und wenn ja wie die Mekoniumausscheidung bei Frühgeborenen in den ersten Lebenstagen unterstützt werden soll. Zur Vorbereitung von Studien zu diesem Thema möchten wir diese Erhebung an Level 1-Perinatalzentren durchführen.

Die Umfrage gliedert sich in drei Abschnitte:
- Allgemeine Angaben
- Nahrungsaufbau, gastrointestinale Komplikationen und mögliche Einflussfaktoren
- Maßnahmen zur Mobilisierung von Mekonium

Die Umfrage besteht aus 13 bis max. 40 Fragen und dauert etwa 5 bis 10 Minuten. Es besteht jederzeit die Möglichkeit, die Umfrage zu unterbrechen und später fortzusetzen.

Herzlichen Dank für Ihre Teilnahme!

Ansprechpartner für Rückfragen und Anmerkungen:
Maximilian Groß
Abteilung für Neonatologie
Universitätsklinik für Kinder- und Jugendmedizin Tübingen
Telefon: 07071 29-80895
E-Mail: maximilian.gross@med.uni-tuebingen.de

Die Teilnahme an dieser Umfrage ist freiwillig. Es werden weder die Arbeitsstätte (Klinik) noch personenbezogene Daten erhoben. Die Daten werden anonym erfasst, eine Zuordnung der Antworten zu den verschickten Teilnahmelinks erfolgt nicht. Die Kontaktdaten (E-Mail-Adressen) der Befragten stammen aus öffentlich zugänglichen Quellen (perinatalzentren.org und die Website des jeweiligen Perinatalzentrums). Die erhobenen Antworten werden sachgerecht zehn Jahre gespeichert, statistisch ausgewertet und anschließend vernichtet. Das Vorgehen wurde mit der Ethikkommission (Projektnummer 012/2022BO2) und den Datenschutzbeauftragten des Universitätsklinikums Tübingen (dsb@med.uni-tuebingen.de) abgestimmt.

Weiter

Befragung unterbrechen

Maximilian Groß, Universitätsklinikum Tübingen, Eberhard Karls Universität Tübingen

DEBUG ▶ Fragebogen [UMF_MEK] – Seite 2 ◀ Seite: ▶ Debug-Informationen ▼

Variable: = ▶

Universitätsklinikum Tübingen

4% ausgefüllt

All Ang Betten GF

Allgemeine Angaben: Anzahl Betten und Fallzahl

A202

Wie viele Betten hat Ihre Neonatologie (Summe aus Neugeborenenstation + Intensivüberwachung + Intensivbehandlung soweit vorhanden)?

- ○ <20
- ○ 20-34
- ○ 35-50
- ○ >50

- ○ Keine Angabe

A203

Wie viele Frühgeborene <1000g Geburtsgewicht werden pro Jahr im Durchschnitt in Ihrer Neonatologie behandelt (als Durchschnitt der letzten fünf Jahre)?

- ○ <5
- ○ 5-24
- ○ 25-50
- ○ >50

- ○ Keine Angabe

Zurück Weiter

Befragung unterbrechen

Maximilian Groß, Universitätsklinikum Tübingen, Eberhard Karls Universität Tübingen

DEBUG ▶ Fragebogen [UMF_MEK] – Seite 3 ◀ Seite: ▶ Debug-Informationen ▼
Variable: = ▶

Universitätsklinikum Tübingen

8% ausgefüllt

Allgemeine Angaben: Berufsgruppe und Berufserfahrung

Allg Angaben Beruf

Welcher Berufsgruppe gehören Sie an?

A204

- ◯ Ärztliche Leitung
- ◯ Oberärztlicher Bereich
- ◯ Fachärztlicher Bereich
- ◯ Assistenzärztlicher Bereich

- ◯ Keine Angabe

Wie viele Jahre Berufserfahrung haben Sie in der Neonatologie?

A205

- ◯ Berufserfahrung in der Neonatologie: [z. B. 7] Jahre

- ◯ Keine Angabe

Zurück Weiter

Befragung unterbrechen

Maximilian Groß, Universitätsklinikum Tübingen, Eberhard Karls Universität Tübingen

DEBUG ▶ Fragebogen [UMF_MEK] – Seite 4 ◀ Seite: ▶ Debug-Informationen ▼
Variable: = ▶

Universitätsklinikum Tübingen

13% ausgefüllt

Angaben zum Nahrungsaufbau Nahrungsaufbau

Machen Sie den Beginn der ersten Milchnahrung vom Zeitpunkt der Mekoniumentleerung abhängig? A305

- ○ Ja
- ○ Nein

- ○ Keine Angabe

Beginnen Sie den enteralen Nahrungsaufbau bei Frühgeborenen <1000g Geburtsgewicht in der Regel innerhalb der ersten 24 Lebensstunden? A301

- ○ Ja
- ○ Nein, später: z. B. erst nach Absetzen von Mekonium

- ○ Keine Angabe

Geben Sie frühestmöglich oral Kolostrum bei Frühgeborenen <1000g Geburtsgewicht (soweit verfügbar)? A302

- ○ Ja, ab dem ersten Tag
- ○ Ja, ab Tag 3 oder später
- ○ Nein

- ○ Keine Angabe

Zurück Weiter

Befragung unterbrechen

Maximilian Groß, Universitätsklinikum Tübingen, Eberhard Karls Universität Tübingen

DEBUG ▶ Fragebogen [UMF_MEK] – Seite 4 ◀ Seite: ▶ Debug-Informationen ▼

Variable: = ▶

DEBUG ▶ Fragebogen [UMF_MEK] – Seite 5 ◀ Seite: ▶ Debug-Informationen ▼
Variable: = ▶

Universitätsklinikum Tübingen

17% ausgefüllt

Angaben zum Nahrungsaufbau

Nahrungsaufbau

A303

Womit beginnen Sie den enteralen Nahrungsaufbau bei Frühgeborenen <1000g Geburtsgewicht in der Regel (soweit verfügbar; Mehrfachnennung möglich)?

- ☐ Muttermilch
- ☐ Spendermilch
- ☐ Frühgeborenenformula
- ☐ Glukose
- ☐ Maltodextrin
- ☐ Andere: z. B. ...

- ☐ Keine Angabe

A304

Ab etwa welchem Lebenstag sind die meisten (>75%) der Frühgeborenen <1000g Geburtsgewicht in Ihrer Neonatologie vollständig enteral ernährt (enterale Zufuhr >140-150 ml/kg/d)?

- ○ Etwa ab dem z. B. 7 Lebenstag

- ○ Keine Angabe

Zurück Weiter

Befragung unterbrechen

Maximilian Groß, Universitätsklinikum Tübingen, Eberhard Karls Universität Tübingen

DEBUG ▶ Fragebogen [UMF_MEK] – Seite 6 ◀ Seite: ▶ Debug-Informationen ▼
Variable: = ▶

Universitätsklinikum Tübingen

21% ausgefüllt

Komplikationen

Gastrointestinale Komplikationen

A401

Wie viele Fälle einer nekrotisierenden Enterokolitis, einer fokalen intestinalen Perforation bzw. eines Mekoniumileus werden in etwa pro Jahr in Ihrer Abteilung beobachtet (als Durchschnitt der letzten fünf Jahre)?

- ☐ Nekrotisierende Enterokolitis: Etwa [Anzahl pro Jahr]
- ☐ Fokale intestinale Perforation: Etwa [Anzahl pro Jahr]
- ☐ Mekoniumileus: Etwa [Anzahl pro Jahr]

- ☐ Keine Angabe

Zurück Weiter

Befragung unterbrechen

Maximilian Groß, Universitätsklinikum Tübingen, Eberhard Karls Universität Tübingen

DEBUG ▶ Fragebogen [UMF_MEK] – Seite 7 ◀ Seite: ▶ Debug-Informationen ▼
Variable: = ▶

Universitätsklinikum Tübingen

25% ausgefüllt

Moegliche Einflussfaktoren

Einsatz von Steroiden und Medikamenten zur Behandlung des Ductus arteriosus Botalli

A501

Verwenden Sie häufig (>75% d. Fälle) systemische Steroide bei Frühgeborenen <1000g Geburtsgewicht in den ersten zwei Lebenswochen, z. B. zur Prophylaxe einer bronchopulmonalen Dysplasie oder bei arterieller Hypotonie (Mehrfachnennung möglich)?

- ☐ Hydrocortison zur Prophylaxe einer bronchopulmonalen Dysplasie
- ☐ Hydrocortison bei arterieller Hypotonie
- ☐ Andere: z. B. andere Steroide oder Indikationen
- ☐ Nein, systemische Steroide kommen nicht oder nur in Einzelfällen zum Einsatz

---

- ☐ Keine Angabe

A502

Welche Medikamente verwenden Sie hauptsächlich zur Behandlung eines hämodynamisch relevanten Ductus arteriosus Botalli (Mehrfachnennung möglich)?

- ☐ Ibuprofen
- ☐ Indometacin
- ☐ Paracetamol
- ☐ Ein Ductus arteriosus Botalli wird bei uns in der Regel nicht medikamentös behandelt

---

- ☐ Keine Angabe

Zurück Weiter

Befragung unterbrechen

Maximilian Groß, Universitätsklinikum Tübingen, Eberhard Karls Universität Tübingen

DEBUG ▶ Fragebogen [UMF_MEK] – Seite 8 ◀ Seite: ▶ Debug-Informationen ▼
Variable: = ▶

Universitätsklinikum Tübingen

29% ausgefüllt

Haeufigkeit Massnahmen

Häufigkeit von Maßnahmen zur Mobilisierung von Mekonium

A601

Kommen in Ihrer Neonatologie Maßnahmen zur Mobilisierung von Mekonium bei Frühgeborenen in den ersten Lebenstagen zum Einsatz?

- ◯ Ja, standardmäßig (auch wenn es nur eine bestimmte Gruppe Frühgeborener betrifft)
- ◯ Bestimmte Maßnahmen werden häufig eingesetzt, jedoch nicht standardmäßig
- ◯ Eher selten und nur in speziellen Fällen
- ◯ Nein, es wird eine spontane Mekoniumausscheidung abgewartet (beendet die Umfrage)

Zurück Weiter

Befragung unterbrechen

Maximilian Groß, Universitätsklinikum Tübingen, Eberhard Karls Universität Tübingen

DEBUG ▶ Fragebogen [UMF_MEK] – Seite 9 ◀ Seite: ▶ Debug-Informationen ▼
Variable: = ▶

Universitätsklinikum Tübingen

33% ausgefüllt

EL und Suppo

Einläufe und Suppositorien zur Mobilisierung von Mekonium

A604

Nachfolgend werden Informationen zu Einläufen und Suppositorien abgefragt. Bitte wählen Sie die entsprechende Option aus, je nachdem ob in Ihrer Abteilung Einläufe und/oder Suppositorien zur Mobilisation von Mekonium eingesetzt werden.

- ○ Wir verwenden Einläufe und/oder Suppositorien zur Mobilisierung von Mekonium
- ○ Wir verwenden *keine* Einläufe und/oder Suppositorien zur Mobilisierung von Mekonium

Zurück Weiter

Befragung unterbrechen

Maximilian Groß, Universitätsklinikum Tübingen, Eberhard Karls Universität Tübingen

DEBUG ▶ Fragebogen [UMF_MEK] – Seite 10 ◀ Seite: ▶ Debug-Informationen ▼
Variable: = ▶

Universitätsklinikum Tübingen

38% ausgefüllt

EL und Suppo Ziel Konzept

Einläufe und Suppositorien: Zielgruppe und Konzept

A625

Bei welchen Frühgeborenen wenden Sie Einläufe und/oder Suppositorien an (Mehrfachnennung möglich, bitte die auf Ihr Vorgehen am ehesten zutreffenden Gruppen ankreuzen)?

- ☐ Bei allen Frühgeborenen
- ☐ <32 Schwangerschaftswochen
- ☐ <28 Schwangerschaftswochen
- ☐ <1500g Geburtsgewicht
- ☐ <1000g Geburtsgewicht
- ☐ Andere: z. B. nur bei hypotrophen Frühgeborenen

- ☐ Keine Angabe

A626

Mit welchem Grundkonzept setzen Sie Einläufe und/oder Suppositorien hauptsächlich ein?

- ○ Eher prophylaktisch (z. B. unabhängig von erfolgter Mekoniumausscheidung oder klinischen Symptomen)
- ○ Eher therapeutisch (z. B. geringe Mekoniumausscheidung und Symptome wie ein aufgetriebenes Abdomen)

- ○ Keine Angabe

Zurück Weiter

Befragung unterbrechen

Maximilian Groß, Universitätsklinikum Tübingen, Eberhard Karls Universität Tübingen

DEBUG ▶ Fragebogen [UMF_MEK] – Seite 11 ◀ Seite: ▶ Debug-Informationen ▼
Variable: = ▶

Universitätsklinikum Tübingen

42% ausgefüllt

EL und Suppo Zusammen

Einläufe und Suppositorien: Zusammensetzung der Einläufe

A606

Was verwenden Sie als Spülflüssigkeit bei Einläufen (Mehrfachnennung möglich)?

- ☐ NaCl 0,9%
- ☐ Glukose 5%
- ☐ Glycerin
- ☐ Aqua dest.
- ☐ Acetylcystein (ACC)
- ☐ Lipidemulsionen
- ☐ Muttermilch
- ☐ Andere bzw. fixe Mischungen aus: z. B. ...

- ☐ Keine Angabe

A607

Verwenden Sie Einläufe mit Röntgenkontrastmittel?

- ○ Ja, regelmäßig
- ○ Selten, nur bei bestimmten Indikationen: z. B. ...
- ○ Nein

- ○ Keine Angabe

A608

Falls Einläufe mit Röntgenkontrastmittel eingesetzt werden: Welche Kontrastmittel kommen bei Ihnen zum Einsatz (Mehrfachnennung möglich)?

- ☐ Osmotisch isotone Röntgenkontrastmittel (z. B. Ultravist®-150)
- ☐ Osmotisch hypertone Röntgenkontrastmittel (z. B. Gastrografin® 100mg/ml)
- ☐ Andere: z. B. ...

DEBUG ▶ Fragebogen [UMF_MEK] – Seite 11 ◀ Seite: ▶ Debug-Informationen ▼

Variable: = ▶

☐ Keine Angabe

Zurück Weiter

Befragung unterbrechen

Maximilian Groß, Universitätsklinikum Tübingen, Eberhard Karls Universität Tübingen

DEBUG ▶ Fragebogen [UMF_MEK] – Seite 12 ◀ Seite: ▶ Debug-Informationen ▼
Variable: = ▶

Universitätsklinikum Tübingen

46% ausgefüllt

Einläufe und Suppositorien: Applikation und Volumen EL und Suppo Applikation

Womit verabreichen Sie Einläufe (Mehrfachnennung möglich)? A610

- ☐ Ernährungssonden
- ☐ Darmrohre
- ☐ Spezielle Einlaufkatheter für Neugeborene
- ☐ Spezielle Spritzenaufsätze, z. B. „Olive“
- ☐ Andere: z. B. ...

- ☐ Keine Angabe

Welche Volumina werden bei Einläufen verabreicht (Mehrfachnennung möglich)? A609

- ☐ Berechnet auf das Körpergewicht: z. B. 10 ml/kg
- ☐ Abhängig von der Zusammensetzung des Einlaufs: z. B. Zusammensetzung und Volumen

- ☐ Keine Angabe

Zurück Weiter

Befragung unterbrechen

Maximilian Groß, Universitätsklinikum Tübingen, Eberhard Karls Universität Tübingen

DEBUG ▶ Fragebogen [UMF_MEK] – Seite 13 ◀ Seite: ▶ Debug-Informationen ▼
Variable: = ▶

Universitätsklinikum Tübingen

50% ausgefüllt

EL und Suppo Haeufigkeit

Einläufe und Suppositorien: Häufigkeit und zeitlicher Rahmen

A605

Ab welchem Zeitpunkt setzen Sie Einläufe und/oder Suppositorien in der Regel ein (Mehrfachnennung möglich)?

- ☐ Ab dem ersten Lebenstag
- ☐ Ab einem bestimmten Lebenstag: z. B. ab dem 3. Lebenstag
- ☐ Wenn bis zu einem bestimmten Zeitpunkt kein/wenig Mekonium ausgeschieden wurde: z. B. kein Mekonium bis Lebenstag 5

- ☐ Keine Angabe

A612

Wie häufig verabreichen Sie Einläufe und/oder Suppositorien (Mehrfachnennung möglich)?

- ☐ Einmal täglich
- ☐ Mehrfach täglich
- ☐ Meist nur ein- bis zweimal, z. B. bis Mekonium ausgeschieden wurde oder im Rahmen einer Kontrastmitteldarstellung
- ☐ Andere Zeitintervalle: z. B. zweimal pro Woche, etc.

- ☐ Keine Angabe

A613

Wie lange verabreichen Sie Einläufe und/oder Suppositorien (Mehrfachnennung möglich)?

- ☐ Bis mindestens einmal Mekonium ausgeschieden wurde
- ☐ Bis zum Auftreten von Übergangsstuhl
- ☐ Bis zum Auftreten von Milchstuhl
- ☐ Bis die Frühgeborenen enteral aufgebaut sind
- ☐ Anderer Zeitpunkt: z. B. innerhalb der ersten Lebenswoche

DEBUG ▶ Fragebogen [UMF_MEK] – Seite 13 ◀ Seite: ▶ Debug-Informationen ▼

Variable: = ▶

Zurück Weiter

Befragung unterbrechen

Maximilian Groß, Universitätsklinikum Tübingen, Eberhard Karls Universität Tübingen

DEBUG ▶ Fragebogen [UMF_MEK] – Seite 14 ◀ Seite: ▶ Debug-Informationen ▼
Variable: = ▶

Universitätsklinikum Tübingen

54% ausgefüllt

Einläufe und Suppositorien: Zusammensetzung der Suppositorien **El und Suppo Suppo**

Welche Zusammensetzung haben die in Ihrer Neonatologie genutzten Suppositorien („Zäpfchen“)? **A611**

- ☐ Glycerin/Glycerol
- ☐ Andere: z. B. ...
- ☐ Wir verwenden keine Suppositorien zur Mobilisierung von Mekonium

- ☐ Keine Angabe

Zurück Weiter

Befragung unterbrechen

Maximilian Groß, Universitätsklinikum Tübingen, Eberhard Karls Universität Tübingen

DEBUG ▶ Fragebogen [UMF_MEK] – Seite 15 ◀ Seite: ▶ Debug-Informationen ▼

Variable: = ▶

Universitätsklinikum Tübingen

58% ausgefüllt

KMO

Orales Röntgenkontrastmittel zur Mobilisierung von Mekonium

A614

Nachfolgend werden Informationen zu oralem Röntgenkontrastmittel abgefragt. Bitte wählen Sie die entsprechende Option aus, je nachdem ob in Ihrer Abteilung orales Röntgenkontrastmittel zur Mobilisierung von Mekonium eingesetzt wird.

- ○ Wir verwenden orales Röntgenkontrastmittel zu Mobilisierung von Mekonium
- ○ Wir verwenden *kein* orales Röntgenkontrastmittel zur Mobilisierung von Mekonium

Zurück Weiter

Befragung unterbrechen

Maximilian Groß, Universitätsklinikum Tübingen, Eberhard Karls Universität Tübingen

DEBUG ▶ Fragebogen [UMF_MEK] – Seite 16 ◀ Seite: ▶ Debug-Informationen ▼
Variable: = ▶

Universitätsklinikum Tübingen

63% ausgefüllt

KMO Zielgru Konzept

Orales Röntgenkontrastmittel: Zielgruppe und Konzept

A627

Bei welchen Frühgeborenen wenden Sie orales Röntgenkontrastmittel an (Mehrfachnennung möglich; bitte die auf Ihr Vorgehen am ehesten zutreffenden Gruppen ankreuzen)?

- ☐ Bei allen Frühgeborenen
- ☐ <32 Schwangerschaftswochen
- ☐ <28 Schwangerschaftswochen
- ☐ <1500g Geburtsgewicht
- ☐ <1000g Geburtsgewicht
- ☐ Andere: z. B. nur bei hypotrophen Frühgeborenen

- ☐ Keine Angabe

A630

Mit welchem Grundkonzept setzen Sie orales Kontrastmittel hauptsächlich ein?

- ○ Eher prophylaktisch (z. B. unabhängig von erfolgter Mekoniumausscheidung oder klinischen Symptomen)
- ○ Eher therapeutisch (z. B. geringe Mekoniumausscheidung und Symptome wie ein aufgetriebenes Abdomen)

- ○ Keine Angabe

Zurück Weiter

Befragung unterbrechen

Maximilian Groß, Universitätsklinikum Tübingen, Eberhard Karls Universität Tübingen

DEBUG ▶ Fragebogen [UMF_MEK] – Seite 17 ◀ Seite: ▶ Debug-Informationen ▼

Variable: = ▶

Universitätsklinikum Tübingen

67% ausgefüllt

KMO Zeitpunkt Haeufi

Orales Röntgenkontrastmittel: Zeitpunkt, Häufigkeit und Typ

A615

Ab welchem Zeitpunkt setzen Sie orales Röntgenkontrastmittel zur Mobilisierung von Mekonium ein (Mehrfachnennung möglich)?

- ☐ Ab dem ersten Lebenstag
- ☐ Ab einem bestimmten Lebenstag: z. B. ab dem 3. Lebenstag
- ☐ Wenn bis zu einem bestimmten Zeitpunkt kein/wenig Mekonium ausgeschieden wurde: z. B. kein Mekonium bis Lebenstag 5

- ☐ Keine Angabe

A616

Wie häufig applizieren Sie orales Röntgenkontrastmittel zur Mobilisierung von Mekonium pro Patienten (Mehrfachnennung möglich)?

- ☐ Einmalig
- ☐ Repetitiv
- ☐ Je nach Indikation: z. B. ...

- ☐ Keine Angabe

A617

Welche oral verabreichten Kontrastmittel kommen bei Ihnen zur Mekoniummobilisation zum Einsatz?

- ☐ Osmotisch isotone Röntgenkontrastmittel (z. B. Ultravist®-150)
- ☐ Osmotisch hypertone Röntgenkontrastmittel (z. B. Gastrografin® 100mg/ml)
- ☐ Andere: z. B. ...

- ☐ Keine Angabe

DEBUG ▶ Fragebogen [UMF_MEK] – Seite 17 ◀ Seite: ▶ Debug-Informationen ▼

Variable: = ▶

Befragung unterbrechen

Maximilian Groß, Universitätsklinikum Tübingen, Eberhard Karls Universität Tübingen

DEBUG ▶ Fragebogen [UMF_MEK] – Seite 18 ◀ Seite: ▶ Debug-Informationen ▼
Variable: = ▶

Universitätsklinikum Tübingen

72% ausgefüllt

MOV

Macrogol (z. B. Movicol®) zur Mobilisierung von Mekonium

A618

Nachfolgend werden Informationen zu Macrogol (z. B. Movicol®) abgefragt. Bitte wählen Sie die entsprechende Option aus, je nachdem ob in Ihrer Abteilung Macrogol zur Mobilisation von Mekonium eingesetzt wird.

- ◯ Wir verwenden Macrogol zur Mobilisierung von Mekonium
- ◯ Wir verwenden *kein* Macrogol zur Mobilisierung von Mekonium

Zurück Weiter

Befragung unterbrechen

Maximilian Groß, Universitätsklinikum Tübingen, Eberhard Karls Universität Tübingen

DEBUG ▶ Fragebogen [UMF_MEK] – Seite 19 ◀ Seite: ▶ Debug-Informationen ▼
Variable: = ▶

Universitätsklinikum Tübingen

76% ausgefüllt

MOV Zielgr Konzept

Macrogol (z. B. Movicol®): Zielgruppe und Konzept

A628

Bei welchen Frühgeborenen wenden Sie Macrogol an (Mehrfachnennung möglich; bitte die auf Ihr Vorgehen am ehesten zutreffenden Gruppen ankreuzen)?

- ☐ Bei allen Frühgeborenen
- ☐ <32 Schwangerschaftswochen
- ☐ <28 Schwangerschaftswochen
- ☐ <1500g Geburtsgewicht
- ☐ <1000g Geburtsgewicht
- ☐ Andere: z. B. nur bei hypotrophen Frühgeborenen

- ☐ Keine Angabe

A631

Mit welchem Grundkonzept setzen Sie Macrogol hauptsächlich ein?

- ○ Eher prophylaktisch (z. B. unabhängig von erfolgter Mekoniumausscheidung oder klinischen Symptomen)
- ○ Eher therapeutisch (z. B. geringe Mekoniumausscheidung und Symptome wie ein aufgetriebenes Abdomen)

- ○ Keine Angabe

Zurück Weiter

Befragung unterbrechen

Maximilian Groß, Universitätsklinikum Tübingen, Eberhard Karls Universität Tübingen

DEBUG ▶ Fragebogen [UMF_MEK] – Seite 20 ◀ Seite: ▶ Debug-Informationen ▼
Variable: = ▶

Universitätsklinikum Tübingen

80% ausgefüllt

MOV Zeit Dauer

Macrogol (z. B. Movicol®): Zeitpunkt und Dauer

A619

Ab welchem Zeitpunkt setzen Sie Macrogol ein (Mehrfachnennung möglich)?

- ☐ Ab dem ersten Lebenstag
- ☐ Ab einem bestimmten Lebenstag: z. B. ab dem 3. Lebenstag
- ☐ Wenn bis zu einem bestimmten Zeitpunkt kein/wenig Mekonium ausgeschieden wurde: z. B. kein Mekonium bis Lebenstag 5

- ☐ Keine Angabe

A620

Wie lange verabreichen Sie Macrogol (Mehrfachnennung möglich)?

- ☐ Bis mindestens einmal Mekonium ausgeschieden wurde
- ☐ Bis zum Auftreten von Übergangsstuhl
- ☐ Bis zum Auftreten von Milchstuhl
- ☐ Bis die Frühgeborenen enteral aufgebaut sind
- ☐ Anderer Zeitpunkt: z. B. innerhalb der ersten Lebenswoche

- ☐ Keine Angabe

Zurück Weiter

Befragung unterbrechen

Maximilian Groß, Universitätsklinikum Tübingen, Eberhard Karls Universität Tübingen

DEBUG ▶ Fragebogen [UMF_MEK] – Seite 21 ◀ Seite: ▶ Debug-Informationen ▼

Variable: = ▶

Universitätsklinikum Tübingen

84% ausgefüllt

Maltodextrin zur Mobilisierung von Mekonium MAL

A621

Nachfolgend werden Informationen zu Maltodextrin abgefragt. Bitte wählen Sie die entsprechende Option aus, je nachdem ob in Ihrer Abteilung Maltodextrin zur Mobilisierung von Mekonium eingesetzt wird.

- ◯ Wir verwenden Maltodextrin zur Mobilisierung von Mekonium
- ◯ Wir verwenden *kein* Maltodextrin zur Mobilisierung von Mekonium

Zurück Weiter

Befragung unterbrechen

Maximilian Groß, Universitätsklinikum Tübingen, Eberhard Karls Universität Tübingen

DEBUG ▶ Fragebogen [UMF_MEK] – Seite 22 ◀ Seite: ▶ Debug-Informationen ▼
Variable: = ▶

Universitätsklinikum
Tübingen

88% ausgefüllt

Maltodextrin: Zielgruppe, Zeitpunkt und Dauer MAL Zielgr Zeitp Dauer

A629

Bei welchen Frühgeborenen wenden Sie Maltodextrin an (Mehrfachnennung möglich; bitte die auf Ihr Vorgehen am ehesten zutreffenden Gruppen ankreuzen)?

- ☐ Bei allen Frühgeborenen
- ☐ <32 Schwangerschaftswochen
- ☐ <28 Schwangerschaftswochen
- ☐ <1500g Geburtsgewicht
- ☐ <1000g Geburtsgewicht
- ☐ Andere: z. B. nur bei hypotrophen Frühgeborenen

- ☐ Keine Angabe

A622

Ab welchem Zeitpunkt setzen Sie Maltodextrin ein (Mehrfachnennung möglich)?

- ☐ Ab dem ersten Lebenstag
- ☐ Ab einem bestimmten Lebenstag: z. B. ab dem 3. Lebenstag
- ☐ Wenn bis zu einem bestimmten Zeitpunkt kein/wenig Mekonium ausgeschieden wurde: z. B. kein Mekonium bis Lebenstag 5

- ☐ Keine Angabe

A623

Wie lange verabreichen Sie Maltodextrin (Mehrfachnennung möglich)?

- ☐ Bis mindestens einmal Mekonium ausgeschieden wurde
- ☐ Bis zum Auftreten von Übergangsstuhl
- ☐ Bis zum Auftreten von Milchstuhl
- ☐ Bis die Frühgeborenen enteral aufgebaut sind

DEBUG ▶ Fragebogen [UMF_MEK] – Seite 22 ◀ Seite: ▶ Debug-Informationen ▼

Variable: = ▶

☐ Keine Angabe

Zurück Weiter

Befragung unterbrechen

Maximilian Groß, Universitätsklinikum Tübingen, Eberhard Karls Universität Tübingen

DEBUG ▶ Fragebogen [UMF_MEK] – Seite 23 ◀ Seite: ▶ Debug-Informationen ▼
Variable: = ▶

Universitätsklinikum Tübingen

92% ausgefüllt

Andere Massnahmen

Andere Maßnahmen zur Mobilisierung von Mekonium

A624

Welche anderen Maßnahmen zur Mobilisierung von Mekonium kommen bei Ihnen – außer den bisher genannten – noch zum Einsatz (Mehrfachnennung möglich)?

- ☐ Orale Gabe von Acetylcystein (ACC)
- ☐ Rektale Stimulation
- ☐ Bauchmassagen
- ☐ Andere Maßnahmen: z. B. ...

- ☐ Keine Angabe

Zurück Weiter

Befragung unterbrechen

Maximilian Groß, Universitätsklinikum Tübingen, Eberhard Karls Universität Tübingen

DEBUG ▶ Fragebogen [UMF_MEK] – Seite 24 ◀ Seite: ▶ Debug-Informationen ▼

Variable: = ▶

Universitätsklinikum Tübingen

96% ausgefüllt

A701

Hier haben Sie die Möglichkeit die Umfrage abzuschließen oder nochmals zum Beginn zurückzukehren, um Ihre Antworten ggf. zu korrigieren.

- ◯ Ich habe die Umfrage abgeschlossen
- ◯ Ich möchte zum Start zurück

Zurück Weiter

Befragung unterbrechen

Maximilian Groß, Universitätsklinikum Tübingen, Eberhard Karls Universität Tübingen

DEBUG ▶ Fragebogen [UMF_MEK] – Seite end Debug-Informationen ▼

Universitätsklinikum Tübingen

Vielen herzlichen Dank für Ihre Teilnahme!

Bei Fragen, Anmerkungen oder Kritik und falls Interesse an einer Rückmeldung zu den Ergebnissen dieser Umfrage besteht, schicken Sie gerne eine E-Mail an maximilian.gross@med.uni-tuebingen.de.

Ihre Antworten wurden gespeichert, Sie können das Browser-Fenster nun schließen.

Maximilian Groß, Universitätsklinikum Tübingen, Eberhard Karls Universität Tübingen
